# Echocardiographic diagnosis of cardiac amyloidosis: Does the masquerader require only a "cherry on top"?

**DOI:** 10.1111/echo.14952

**Published:** 2020-11-30

**Authors:** Anatoli Kiotsekoglou, Samir K. Saha, Navin C. Nanda, Per Lindqvist

**Affiliations:** ^1^ Department of Clinical Physiology Umeå University Hospital Umeå Sweden; ^2^ Cardiology Division University of Alabama at Birmingham Birmingham AL USA

Cardiac involvement of amyloidosis can be challenging to diagnose because of the varying clinical and imaging manifestations. When present, the disease may mimic a variety of other cardiac illnesses, such as hypertrophic cardiomyopathy, left ventricular hypertrophy of any cause, heart failure with preserved ejection fraction, or hypertensive heart disease. Cardiac amyloidosis may even coexist with aortic stenosis^1^ and other patients with new‐onset atrial fibrillation.^2^ In this editorial, we discuss the role of left ventricular speckle‐tracking echocardiography in the assessment of cardiac amyloidosis (CA) in reference to the original work published in this issue of the journal.^3^

Two‐dimensional strain imaging of cardiac chambers has further advanced the field of echocardiography in terms of reproducible assessment of cardiac mechanical function different from the traditional estimation of left ventricular ejection fraction. One of the most intriguing discoveries in CA is the unraveling of the existence of a "cherry‐like" strain preservation pattern in the left ventricular apex (compared with other segments) with an extraordinarily high degree of spatial resolution. By taking the ratio of apical longitudinal strain versus basal and mid‐longitudinal strain, a value of over 1.3 predicted the existence of CA.^4^ With the presence of the "cherry‐like" apical sparing strain associated with other echo features such as increased left and right ventricular (LV, RV) thickness, atrial enlargement, restrictive LV filling pattern, and pericardial effusion, suspicion and a "red flag" for CA can be raised. Such a high degree of suspicion would motivate further investigation using other more accurate diagnostic modalities such as bone tracer scintigraphy, cardiac magnetic resonance imaging (CMR), and screening for monoclonal gammopathy (AL amyloidosis).

The basis of the study^3^ the editorial is focusing on is the data previously obtained from a group of 181 consecutive patients with the mixed disease and clinical suspicion of CA aged ≥ 70 years, who underwent 99 mTc‐PYP scintigraphy at the Kumamoto University Hospital, Kumamoto, Japan, between January 2012 and December 2018.^5^ A logistic regression analysis of all clinical and echocardiographic parameters revealed that high‐sensitivity cardiac troponin T (hs‐cTnT) ≥ 0.0308 ng/mL, left ventricular posterior wall thickness ≥ 13.6 mm, and a wide QRS complex (QRS ≥ 120 ms) were strongly associated with 99 mTc‐PYP positivity. The investigators of the latter study representing the Kumamoto University Hospital, Japan, proposed a new index (the Kumamoto criteria) for predicting 99 mTc‐PYP positivity by adding 1 point for each of the 3 variables. The 99 mTc‐PYP–positive rates increased by a factor of 4.57 for each 1‐point increase (*P* < .001). Zero points corresponded to a negative predictive value of 87%, and three points corresponded to a positive predictive value of 96% for 99 mTc‐PYP positivity. The authors have convincingly shown that as many as 96% of the patients with all the three variables present (a score of three) had positive scan results using the tracer. In contrast, only 13% of the subjects with a score of zero had a positive scan. In the latter group of subjects, other factors such as TTR mutations and carpal tunnel syndrome could help diagnose CA.

In the study published in this journal,^3^ the authors (also from the Kumamoto University, Japan) wanted to explore whether additional examinations, including echocardiographic assessment of myocardial strain using two‐dimensional speckle‐tracking echocardiography, could increase the pretest probability of 99mTc‐PYP scintigraphy for these patients, particularly in subjects with low scores (zero to one) on the basis of the Kumamoto criteria.^5^ They quantified left ventricular segmental strain in the basal, mid‐, and apical regions in order to calculate an index which represents a ratio of the apical versus a combination of basal and mid‐ventricular strain, using the classic Bull's eye pattern of strain distribution (the so‐called "cherry‐shaped" apical strain pattern), along the entire 17 left ventricular segments. The index was termed RapLSI (relative apical sparing longitudinal index). The data of their study showed that in the finally included 109 subjects, 90 had negative scan results, and 19 subjects had positive scan results. However, only a handful of measures differed between the two groups: The prevalence of carpal tunnel syndrome was higher in the positive scan group, LV wall thicknesses were greater, and the RapLSI ratio was also greater in the same group with a positive scan. The authors concluded that the apex‐to‐mid‐basal LV longitudinal strain ratio might increase the pretest probability of ^99m^Tc‐PYP scintigraphy in patients suspected to have CA.

The pathophysiological basis of the apical sparing is not fully understood. Late gadolinium enhancement by CMR^6^ and SPECT imaging using technetium 99m pyrophosphate have shown evidence of preferential deposition of amyloid in the basal and mid‐LV segments, which would explain the phenomenon of "cherry on the top" observed by speckle‐tracking echocardiography in Bull's eye configuration.^7^ However, Bravo et al,^8^ in a contradictory study, demonstrated no significant base‐to‐apex gradient in the tissue concentration or proportion of amyloid infiltration. Rather, the authors argued that the segmental distribution (not the gradient) of the total amyloid burden explained the phenomenon of apical sparing. They used 18F‐florbetapir PET imaging to arrive at this conclusion in a small series of subjects with light‐chain CA. Whatever may be the reason, the cherry‐like apical sparing by itself is insufficient to make a definitive diagnosis of CA.

It is undeniable that advanced imaging using SPECT, PET, and CMR, along with cardiac biopsies, is needed to make an accurate final diagnosis of CA. However, bedside echocardiography, using a combination of standard and speckle‐tracking imaging, may provide a reasonable, noninvasive method aiming to raise suspicion for CA. Table 1 underscores the importance of multiparametric echo data to arrive at a possible diagnosis of CA sufficient to refer for additional imaging, such as a bone scan.

**Table 1:** Echocardiographic measures raising suspicion for cardiac amyloidosis

Measures	Values	Authors (references)
Apex‐to‐base left ventricular strain ratio	>2,5	Senapati, et al^4^
Diastolic dysfunction	At least grade 2	Fazlinezhad and Naqvi ^15^
RV apical sparing with RV apical free wall strain	>27%	Arvidsson, et al^11^
Right atrial reservoir strain (3D speckle‐tracking echo strain)	>24%	Nemes, et al^16^
Left atrial reservoir strain	<29%	Henein, et al^12^
E/E´ ratio	>14	
Left ventricular posterior wall thickness	> 13.6 mm	Marume, et al^5^
Left ventricular septal wall thickness	>16 mm	Arvidsson, et al^11^

Note

Abbreviations: 3D = three‐dimensional; E/E’ = mitral pulsed‐Doppler early inflow wave velocity/tissue Doppler early inflow wave velocity; RV = right ventricular.

In a large registry‐based study involving patients from the United Kingdom and Italy, two different sets of echocardiographic scorings were used: One is the amyloid, AL score, and the other one is the increased wall thickness, IWT score. The echo measures included relative wall thickness (RWT), tricuspid annular plane systolic excursion (TAPSE), E‐wave/e’‐wave (E/e') ratio, and longitudinal strain (LS %) for the AL score. RWT, TAPSE, E/e', LS %, and septal longitudinal systolic apex‐to‐base ratio (SAB) were chosen for the IWT score. For the AL score, RWT and E/e' ratio were assigned two points, while TAPSE and LS % were given one point each. For the IWT score, RWT and SAB were given three points each, and TAPSE was graded for two points, while E/e' ratio and LS % were given one point each. For the systemic AL amyloidosis, a score of > 3 yielded an optimal sensitivity and specificity (83% and 85%, respectively). For the increased wall thickness group, a score of > 5 had corresponding sensitivity and specificity values of 82% and 72%, respectively.^9^ Two additional measures used were the left atrial area and the LV ejection fraction/global strain ratio that were useful to quantify the disease burden based on extracellular volume estimation by MRI.

It is quite intriguing to mention here that a much simpler form of a scoring system may also yield excellent sensitivity and specificity to distinguish AL from hypertrophic cardiomyopathy. Indeed, Gustavsson and colleagues have shown that an interventricular septal/posterior wall thickness ratio of < 1.6 in combination with a summed QRS voltage of < 30 mm could detect ATTR with a sensitivity of 94% and a specificity of 83%, while using these thresholds as diagnostic cutoffs.^10^ The same research group has also shown an exaggerated right ventricular apical strain in ATTR amyloidosis in contrast to those with hypertrophic cardiomyopathy.^11^ We may designate this as the Umea criteria.

**Figure 1:**
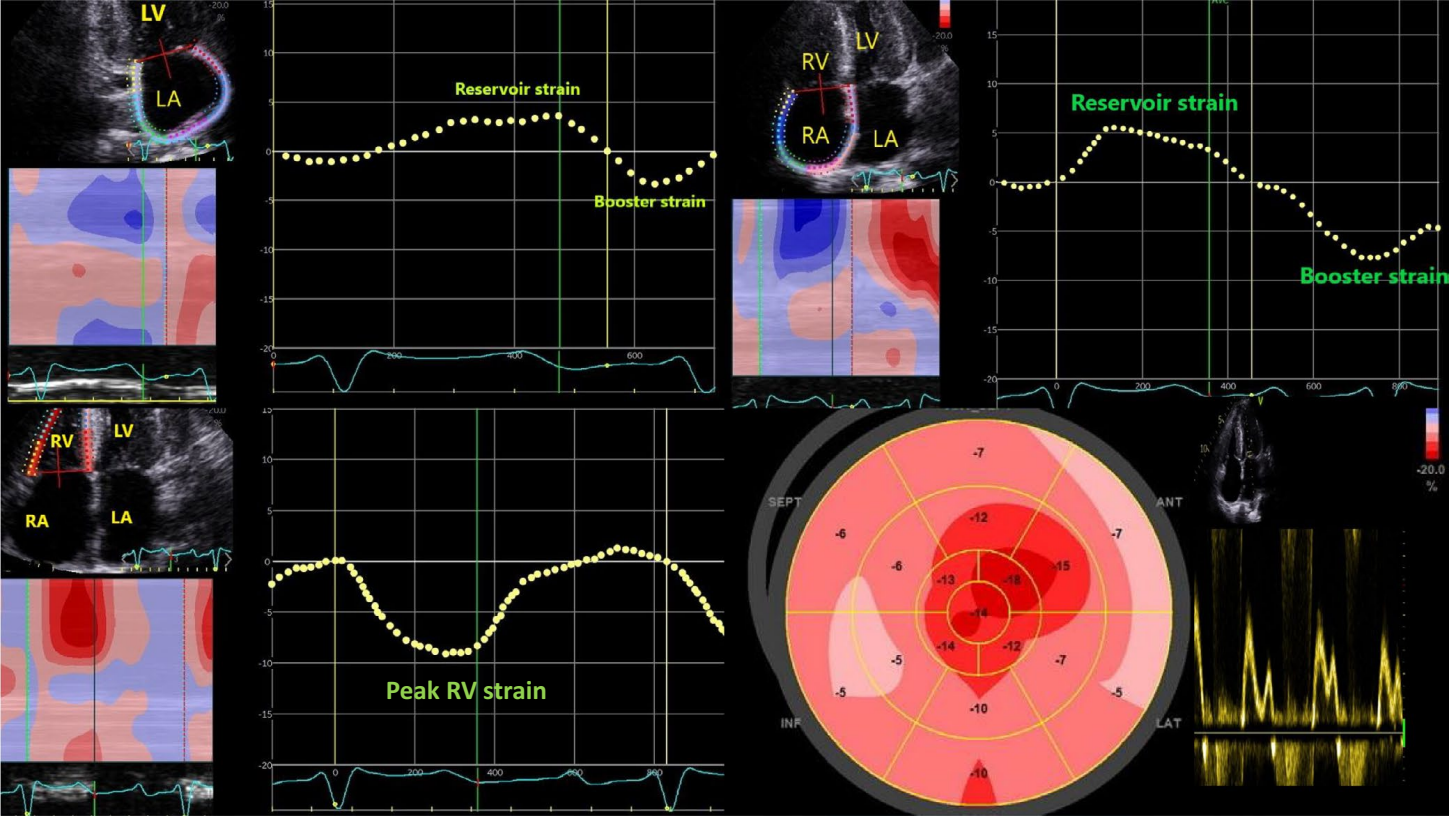
Four‐chamber strain imaging using speckle‐tracking echocardiography in a patient with biopsy‐verified light‐chain amyloidosis. Upper left: atrial (LA) strain showing reservoir and booster components. Upper right: similar phasic strain graphs of the right atrium (RA). Lower left: right ventricular (RV) peak systolic strain. Lower right: left ventricular (LV) global longitudinal strain with a "cherry‐on‐top" strain disposition on Bull's eye pattern of distribution of LV segmental strain and mitral E/A ratio showing pseudonormal LV filling pattern in the same patient

Like a host of cardiac illnesses, left atrial (LA) deformation may also be diminished in ATTR amyloidosis. Henein et al^12^ have shown that LA reservoir strain during ventricular systole is reduced in AL even in the absence of LA enlargement. As shown in Figure 1, bi‐atrial and right ventricular strain may provide useful diagnostic measures complementing the standard echo‐Doppler data. All the suggested cardiac comorbidities associated with CA, such as aortic stenosis,^1^ atrial fibrillation,^2^ and heart failure with preserved ejection fraction, have been shown to have diagnostic values using bi‐atrial and right ventricular strain.^13^

To conclude, we are pleased that Usuku and colleagues have explored the utility of Kumamoto criteria in CA. The authors of this editorial would like to propose that larger multicenter studies are probably necessary to form a universal consensus, based on all the available published data, including the Umea and Kumamoto criteria. This would make clinical assessment and management of cardiac amyloidosis simpler and cost‐effective and avoid misdiagnosis of this life‐threatening disease.^14^
